# Effects of strontium ions with potential antibacterial activity on in vivo bone regeneration

**DOI:** 10.1038/s41598-021-88058-1

**Published:** 2021-04-22

**Authors:** Nafiseh Baheiraei, Hossein Eyni, Bita Bakhshi, Raziyeh Najafloo, Navid Rabiee

**Affiliations:** 1 Tissue Engineering and Applied Cell Sciences Division, Department of Anatomical Sciences, Faculty of Medical Sciences, Tarbiat Modares University, Tehran, Iran; 2 Department of Anatomical Sciences, Faculty of Medical sceinces, Tarbiat Modares University, Tehran, Iran; 3 Department of Bacteriology, Faculty of Medical Sciences, Tarbiat Modares University, Tehran, Iran; 4 Department of Bio-Informatics, Faculty of Interdisciplinary Science and Technology, Tarbiat Modares University, Tehran, Iran; 5 Department of Chemistry, Shahid Beheshti University, Tehran, Iran

**Keywords:** Biotechnology, Cell biology, Chemistry, Materials science

## Abstract

Bioactive glasses (BGs) have attracted added attention in the structure of the scaffolds for bone repair applications. Different metal ions could be doped in BGs to induce specific biological responses. Among these ions, strontium (Sr) is considered as an effective and safe doping element with promising effects on bone formation and regeneration. In this experiment, we evaluated the antibacterial activities of the gelatin-BG (Gel-BG) and Gel-BG/Sr scaffolds in vitro. The osteogenic properties of the prepared scaffolds were also assessed in rabbit calvarial bone defects for 12 weeks. Both scaffolds showed in vivo bone formation during 12 weeks with the newly formed bone area in Gel-BG/Sr scaffold was higher than that in Gel-BG scaffolds after the whole period. Based on the histological results, Gel-BG/Sr exhibited acceleration of early-stage bone formation in vivo. The results of antibacterial investigation for both scaffolds showed complete growth inhibition against *Escherichia coli* (*E. coli*). Although Gel-BG revealed no antibacterial effect on *Staphylococcus aureus* (*S. aureus*), the Gel-BG/Sr was able to partially inhibit the growth of *S. aureus*, as detected by threefold reduction in growth index. Our results confirmed that Sr doped BG is a favorable candidate for bone tissue engineering with superior antibacterial activity and bone regeneration capacity compared with similar counterparts having no Sr ion.

## Introduction

Almost 1.3 million people experience bone graft surgeries due to the skeletal defects made by either accidents or diseases annually in the United States^1^. Obesity, genetic abnormalities, increased rate of accidents as well as the aging population are all considered as reasons which increase the number of bone lesions around the world^2^. Osteoporosis generated by decreased bone mineral density influences more than 200 million people worldwide, with half of this population undergoing a minimum one fracture during their lifetime^3^. Bone grafting either autografts or allografts are associated with limitations such as additional surgery, potential risk of transmitting diseases, immunological response, and long period issues^4^. Therefore, there is still a demand for developing safer and more effective alternatives. Bone tissue engineering is a promising strategy that aims to fabricate interconnected porous graft substitutes for bone defects reconstruction. Among material used for synthetic bone scaffolds, bioactive glasses (BGs) have attracted more attention in the structure of bone repair scaffolds in many investigations due to the properties such as osteogenesis, high level of bioactivity as well as the ability to bond with soft and hard tissues^5,6^. The synergistic effects of Si, Ca and P ions released from BG, could promote differentiation of osteoblasts via activation of osteogenesis-related signaling pathways^7^. Magnetic BGs are also reported having potential for hyperthermia treatment of malignant tumors, including bone cancer^8^. Interestingly, BGs have been clinically used to treat damages made by periodontal disease^9^ as well as for ocular surgery applications^10^.

Different metal ions could be doped in BGs to induce specific biological responses. Among these ions, strontium (Sr) is an alkaline earth metal which is presently utilized for the treatment of osteoporosis^11^. Strontium renelate has also been stated to decrease the rate of fractures in elderly patients having osteoporosis^12^ and has been clinically used to treat osteoporosis in postmenopausal patients^13^. Biomaterials containing Sr have been proved to enhance bone formation or/and remodeling^14^. Also, Sr is considered as an effective and safe doping element which its effect on bone formation and remodeling becomes more noticeable and different over time depending on the applied concentration^14^. This ion is reported to accelerate osteogenesis^15^ and mineralization, as well^16^. The effects of Sr on bone healing and regeneration have been extensively studied in vitro and in vivo^17–19^. For example, Sr containing BG microspheres (Sr-BGM) have been shown to significantly improve early angiogenesis via modulating macrophages towards the M2 phenotype expressing a great value of platelet-derived growth factor-BB (PDGF-BB). The authors assumed that this early vascularization could efficiently promote new tissue regeneration, including bone formation^20^. Also, osteogenic capability of thermosensitive p (N-Isopropylacrylamide-co-butyl Methylacrylate) hydrogel (PIB nanogel) was increased significantly following the addition of mesoporous bioactive glass containing Sr (Sr-MBG). Scaffolds were inserted in rat femur defect two months after making the osteoporosis model. PIB nanogel was considered an excellent carrier for primary osteoblasts, which, together with Sr-MBG, improved the regeneration of the produced femur defects synergistically^3^. Addition of Sr and, or Li have also been shown to alter physicochemical properties of BG porous scaffolds, promoting osseointegration and bone remodeling in a rabbit femoral defect model^6^. The simultaneous effect of applying Sr and Co ions on the acceleration of bone healing and vascularization was further confirmed by implanting Sr-Co-BG seeded with human umbilical cord perivascular cells (HUCPVCs) in the knee defect of the rabbits for 12 weeks. The results revealed significant improved angiogenic and regeneration potential of BGs after being doped with Sr and Co^21^. Previously, our group demonstrated that BG/Sr containing scaffolds promote proliferation and osteogenic differentiation of Mesenchymal Stem Cells (MSCs) as well as angiogenesis^17^. Here, we further evaluated the osteogenic properties of the gelatin-BG/Sr scaffolds in rabbit calvarial bone defects as well as their antibacterial features. The obtained results were compared with those without Sr.

## Materials and methods

### Materials

All materials were purchased from Sigma- Aldrich (Germany) unless otherwise is specified.

### Scaffold fabrication

Bioglass (BG) based on the CaO– SiO_2_– Na_2_O –P_2_O_5_ system and BG having strontium (BG/Sr) in a SiO_2_–CaO–SrO–P_2_O_5_ system was fabricated according to our previous protocol by sol- gel method with Sr was substituted for Ca at the percentage of 5 wt%^17^. Scaffolds were prepared via freeze drying method, as previously explained^17^. Briefly, 15% w/v of the synthesized BG powder was added to the 5% (w/v) aqueous solution of gelatin (Gel) and the obtained solution was then cast in a Teflon mold and frozen at – 20 °C and − 80 °C for 5 and 12 h, respectively. The frozen samples were then lyophilized in a freeze drier (Alpha 1–4 LDplus, Martin Christ, Germany) for 48 h to fabricate porous scaffolds. The same instructions were used with BG/Sr powder to make the Gel- BG/Sr scaffolds (15% w/v). Samples were cross linked using 0.5% glutaraldehyde for 24 h, followed by excessive washing in deionized water for three days and further lyophilization.

#### Scaffold characterization

The morphology and microstructure of the scaffolds were observed by scanning electron microscopy (SEM; XL30, Philips).

### Investigational animals and surgical procedures

Animal investigations were approved by the Ethics Committee of Tarbiat Modares University, Iran (IR.MODARES.REC.1398.070). All methods were performed in accordance with the relevant guidelines and regulations. Twelve New Zealand white rabbits of 2.6–3 kg in weight were used and randomly divided into three investigational groups (n = 4/group). A standard environment such as humidity, temperature, 12/12 h light/dark, and standard food and water were applied before and after surgery. General anesthesia was accomplished with Xylazine (10 mg/Kg, 2%, Alfasan, Woerden-Holland) and Ketamine (90 mg/Kg, 10%, Alfasan, Woerden-Holland). Rabbits were prepared and draped using povidone iodide to sterilize the area. The calvaria were exposed by a skin incision (3 cm incision along the midline of the scalp) and then, muscle and periosteum were resected. Three standardized defects (8 mm in diameter) were formed in the parietal bones with low rotation speed of surgical trephine using sterile saline buffer to cool while clearing any residual debris. Two defects were filled with Gel-BG/Sr and Gel-BG scaffold, and the third one was left unfilled as a control group. Attention was taken to avoid displacement of the scaffolds into the other defects. At the end of the surgery, periosteum, muscle, and skin were repositioned and closed with absorbable VICRYL (Johnson & Johnson Co., USA) sutures and Tetracycline (Iran Darou, Iran) was sprayed on calvaria. Amoxicillin (0.1 ml/kg, 15%, Tolide Darou, Iran) was also administrated intramuscularly to avoid infections. Animals were safe and kept warm in separate cages until recovery. They were then guided to the holding room and ordered Ketorolac Tromethamine (Tarasyn, Korea) for three days to control postoperative pain.

### Histological analysis

Rabbits were sacrificed at four, eight, and 12 weeks after surgery, and calvaria were taken for macroscopic and microscopic studies. Samples were fixed in 10% neutral buffered formalin followed by decalcification via immersing in 10% v/v nitric acid for 14 days (being refreshed every 48 h). Then, dehydration was performed in a graded series of ethanol (80–100%). Each sample was embedded in paraffin and sectioned (5 microns) by microtome (Leica Microsystems SP 1600, Nussloch, Germany) for Alizarin Red, Hematoxylin & Eosin (H&E) and Masson’s Trichrome staining evaluations. At least, three histological sections were selected and investigated using a light microscope (Leica Microsystems AG, Wetzlar, Germany). All histomorphometric data were obtained from Image J software (NIH, Maryland, USA) to evaluate the new bone, residual graft, and connective tissue (n = 6 sections/ each group).

### In vitro antibacterial activity assay

Scaffolds were powdered, sterilized with Ultra Violet (UV) and added to 2 ml Nutrient Broth (NB) bacterial culture medium at a final concentration of 175 mg/mL. Each sample suspension was incubated at 37℃ for 48 h under aerobic conditions, after which the samples were centrifuged (for 5 min at 15,000 rpm). One ml from the supernatant was transferred to sterilized tubes, and separate tubes were inoculated with each of the test microorganisms (*Staphylococcus aureus* ATCC28923 and *Escherichia coli* ATCC28922) at a final concentration of 10^6^ CFU/mL. Free NB served as negative control, and bioglass free NB culture of *S. aureus* and *E. coli* were used as positive controls. The inoculated samples were incubated at 37̊C under aerobic conditions for an overnight after which samples were serially diluted and an aliquot of 100 µL was cultured on Mueller–Hinton agar (MH) at 37 °C for 24 h. The total viable count was performed and the Colony Forming Unit (CFU) was determined on MH plates. The inhibition of growth was calculated as the logarithmic reduction in colony counts on MH plates.

### Statistical analysis

All data were evaluated using the ANOVA test of SPSS (version 12.0.1, Chicago, USA). Differences were considered significant at *P* values ≤ 0.05.

## Results

### Macroscopic and histologic evaluation

**Figure 1:**
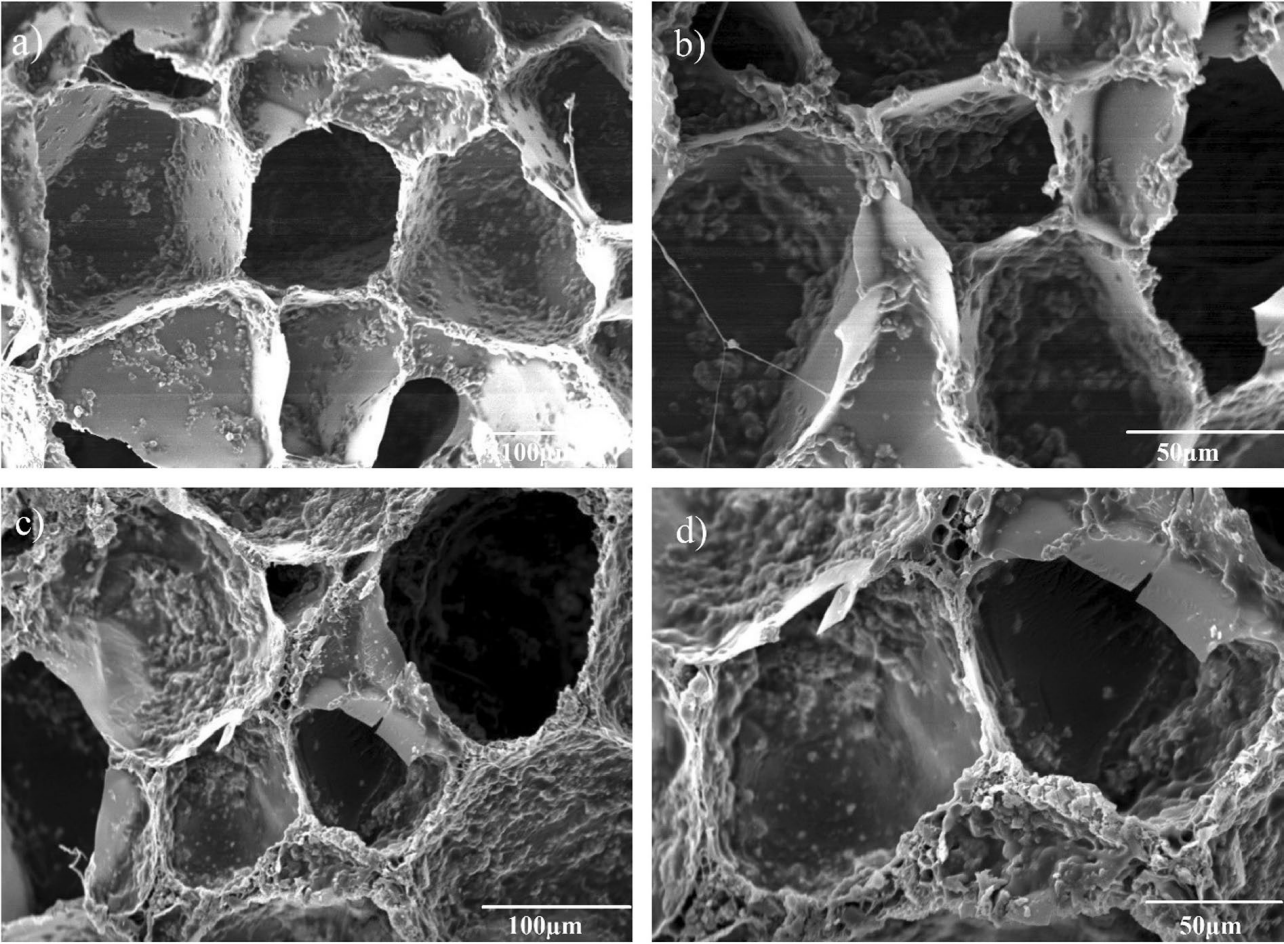
SEM images of the prepared scaffolds with different magnifications: Gel-BG (**a**,**b**), Gel-BG/Sr (**c**,**d**).

In the first step, in order to evaluate the morphology and surface topography of the scaffolds, SEM was performed. As can be seen in Fig. 1, SEM images shows interconnected porosities with the average pore diameter in the range of 80–200 µm. The presence of BG powder is obviously observed on the surface of both scaffolds with more inhomogeneous roughness for Sr containing scaffolds.

**Figure 2:**
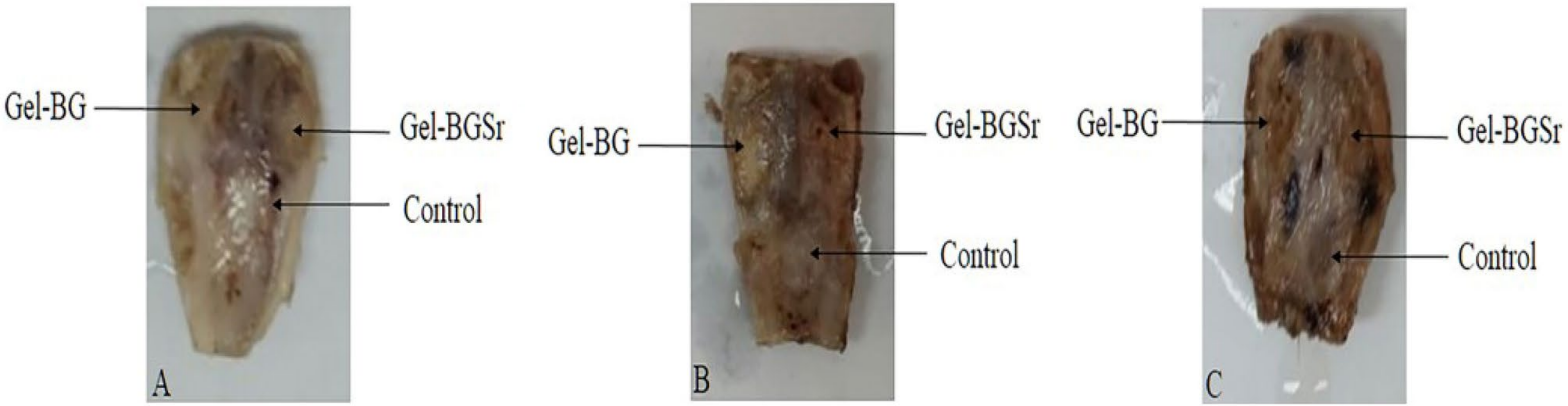
Macroscopic vault appearance of the defects, showing Gel-BG/Sr and Gel-BG scaffolds and Control (unfilled) after (**A**) 4; (**B**) 8 and (**C**) 12 weeks.

**Figure 3:**
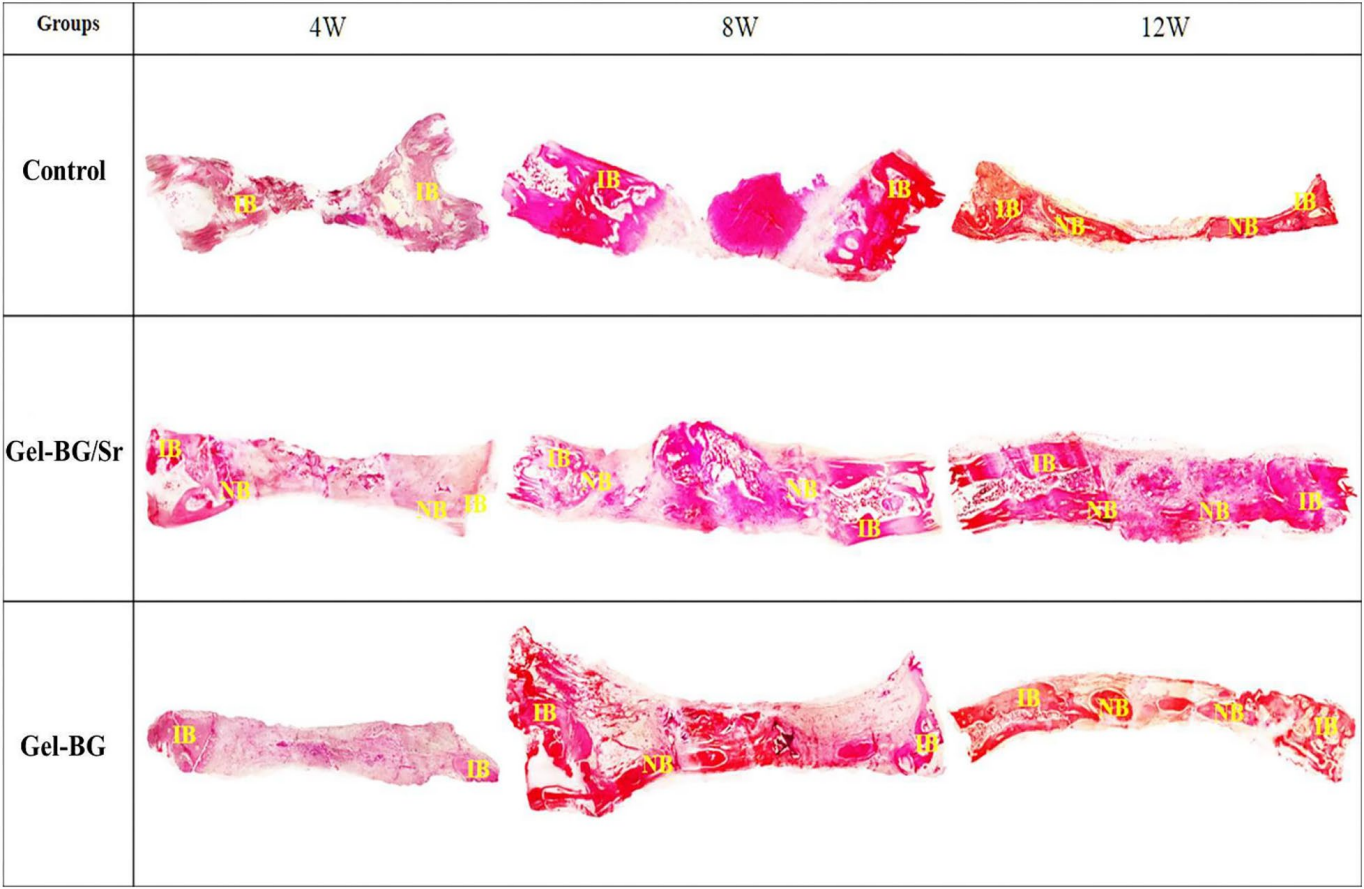
Histological examination (H&E staining) at 4, 8 and 12 weeks. IB (Intact Bone), NB (New Bone) (magnification ×40).

**Figure 4:**
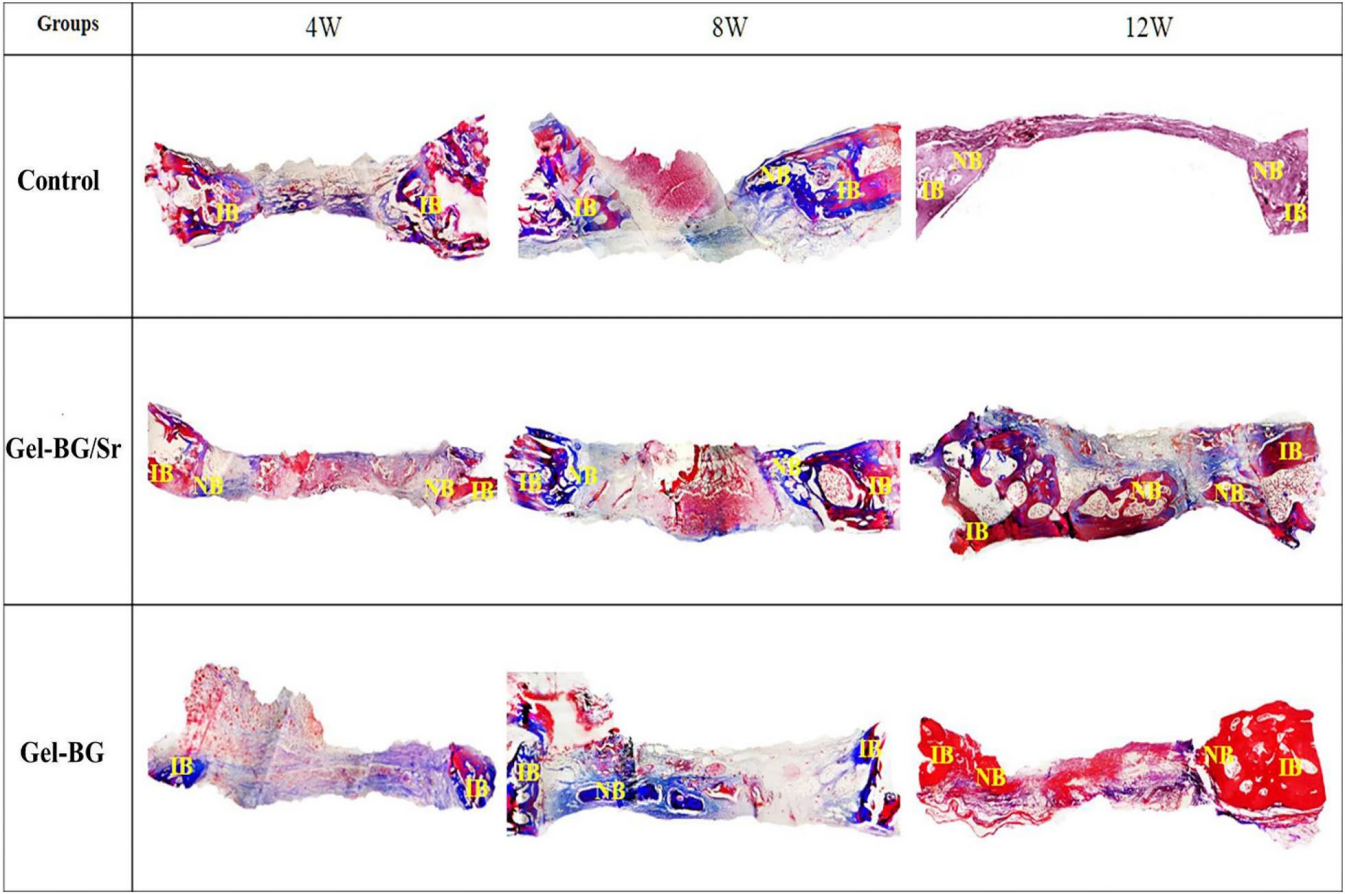
Histological examination (Trichorm-Masson staining) at 4, 8 and 12 weeks. IB; Intact Bone, NB; New Bone (magnification ×40).

Also, the osteogenic capability of the prepared scaffolds was investigated by implanting Gel-BG/Sr and Gel-BG disks into 8 mm full-thickness calvarial defects. This procedure was conducted after 4, 8 and 12 weeks. To do this experiment, rabbits were sacrificed with an overdose of anesthetics and the cranial was carefully removed. Macroscopically, the entire defect area was covered with the fibrous scar and no inflammation was observed in all experimental groups. Defects treated with scaffolds appeared thinner than the surrounding bone 12 weeks after implantation. However, those that had Gel-BG/Sr were contiguous, being hardly detectable from the nearby bone tissue with no movement (Fig. 2). For H&E staining investigation, the samples were studied with a light microscope at magnifications of ×40 and ×100. Defects filled with the Gel-BG/Sr scaffolds presented woven bone development for four weeks. New bone creation was noted to begin bridging the defects at eight weeks, with noticeable cell migration. The border of the defects was hardly distinguishable from the nearby bone tissue. Although no noticeable cell permeation or new bone formation was observed in Gel-BG treated defects at four weeks, cell impregnation, new bone formation, and bridging in this group was started at eight weeks, which then were increased after twelve weeks. The unfilled defects (control group) did not heal completely, and the new bone was only noted at the defect margin with the fibrous tissue covered the rest of the defect (Fig. 3). Additionally, Masson’s trichrome staining was used to confirm new bone formation (Fig. 4). For Gel-BG/Sr scaffolds, the displacement of the mature collagen at the new bone formation zone was detected. Woven bone formation was observed at four weeks, followed by the newly bone creation after eight and 12 weeks. Unlikely, in defects contained Gel-BG scaffolds as well as the control group, woven bone formation was not detected at four weeks. In defects treated with scaffolds, the newly produced bone was observed on each lateral of the margin defects as well as between the implanted scaffolds. Although in all experimental groups, the Masson’s trichrome images showed the development of new collagen fibers (blue color), in the defects filled with Gel/BG-Sr, the number of synthesized collagen fibers was higher compared to other groups following 12 weeks post-implantation.

**Figure 5:**
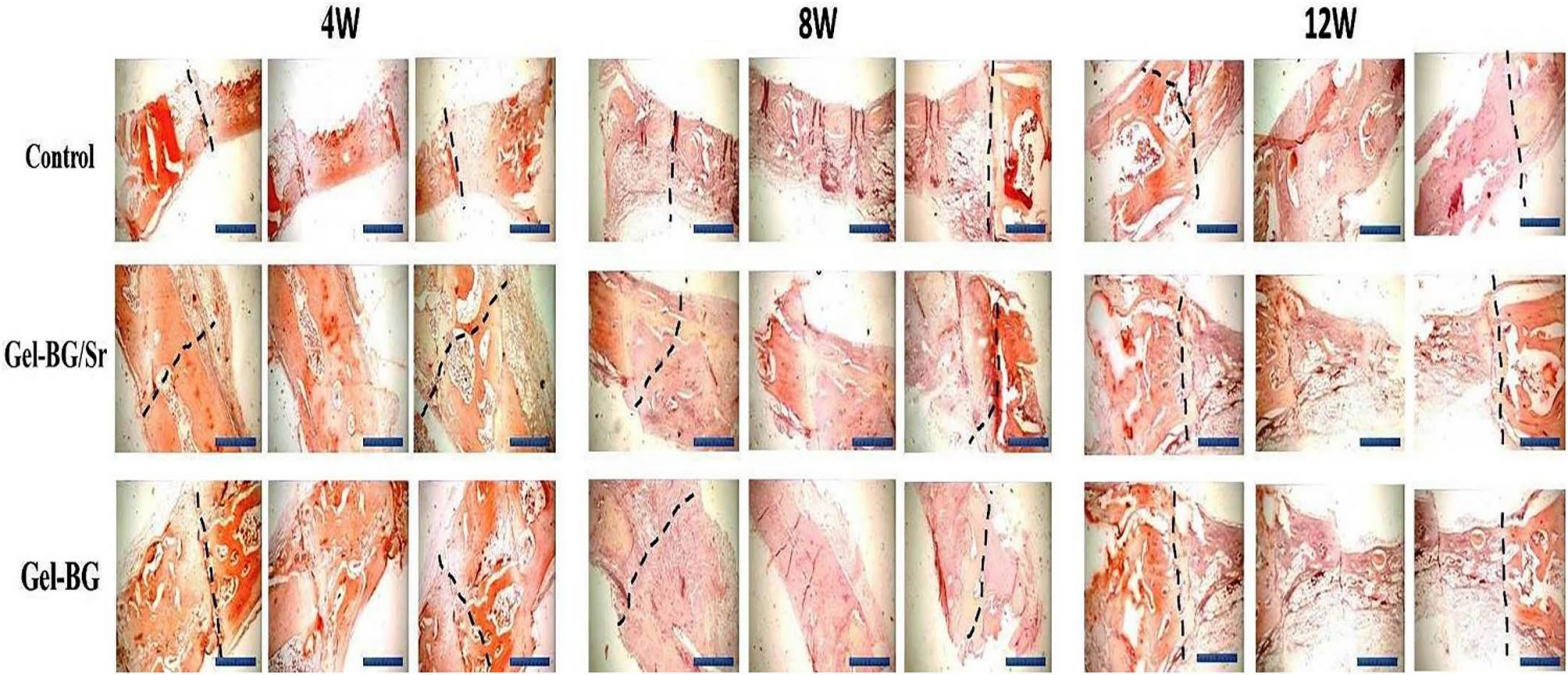
Histological examination (Alizarin red staining), showing the stages of mineralization of formed tissue after implantation of scaffolds at 4, 8 and 12 weeks. The dotted line indicates the border between intact bone and scaffolds (Scale bar: 10 mm).

Mineralization stages of the formed tissue were observed by Alizarin Red staining at specific time points. As can be seen in Fig. 5, woven bone development was detected in Gel-BG/Sr at four weeks, followed by considerable new bone formation and bridging at eight and 12 weeks. For the Gel-BG scaffold, newly bone development was little, and most of the defects were clear. The new bone was only noted at the margin with fibrous tissue was observed in the rest of the defect for the unfilled defect. Our findings showed that implantation of Gel-BG/Sr could upgrade bone regeneration more successfully than Gel-BG in this model.

### Histomorphometric analysis

The results of the histomorphometric analysis is presented in Table 1 during four, eight, and 12 weeks. As can be seen, the percentage of new bone was 49.10 ± 1.03%, 33.88 ± 0.35%, and 17.32 ± 0.36% for Gel-BG/Sr, Gel-BG, and control groups at 12 weeks, respectively (Table 1). Bone healing was significantly increased in Gel-BG/Sr compared with other groups at 12 weeks (*P ≤ 0.05). The residual graft was, also, gradually decreased in Gel-BG/Sr and Gel-BG groups reaching 41.50 ± 0.20% and 51.34 ± 0.06%, respectively. Also, the amount of connective tissue of composite scaffolds (Gel-BG/Sr and Gel-BG) was gradually decreased during 12 weeks, while the corresponding data changes were not apparent for the unfilled groups.

**Table 1:** Histomorphometric analysis of new bone, connective tissue and residual graft during 4, 8 and 12 weeks.

	Gel-BG/Sr	Gel-BG	Control
**NB (%)**			
4 weeks	11.71 ± 0.84^a^	7.1 ± 0.7^b^	4.2 ± 0.42^c^
8 weeks	30.2 ± 3.3^a^	21.02 ± 0.88^b^	14.15 ± 0.27^c^
12 weeks	49.1 ± 3.73^a^	33.88 ± 0.35^b^	17.32 ± 0.36^c^
**RG (%)**			
4 weeks	60.68 ± 1.62^a^	69.8 ± 0.54^b^	0.00^c^
8 weeks	53.56 ± 1.64^a^	58.43 ± 1.41^b^	0.00^c^
12 weeks	41.50 ± 1.94^a^	51.34 ± 0.98^b^	0.00^c^
**CT (%)**			
4 weeks	28.15 ± 0.73^a^	25.10 ± 0.3^b^	95.08 ± 0.21^c^
8 weeks	16.23 ± 0.41^a^	20.55 ± 0.68^b^	85.85 ± 0.56^c^
12 weeks	9.4 ± 0.18^a^	14.78 ± 0.09^b^	82.68 ± 0.38^c^

Data are represented as mean ± SD. Different letters in each row show P < 0.001; a, b, c: significant difference with other groups at the same time; *NB* The percentage of New Bone, *RG* the percentage of Residual Graft, *CT* The percentage of Connective Tissue.

### Antibacterial investigations

**Figure 6:**
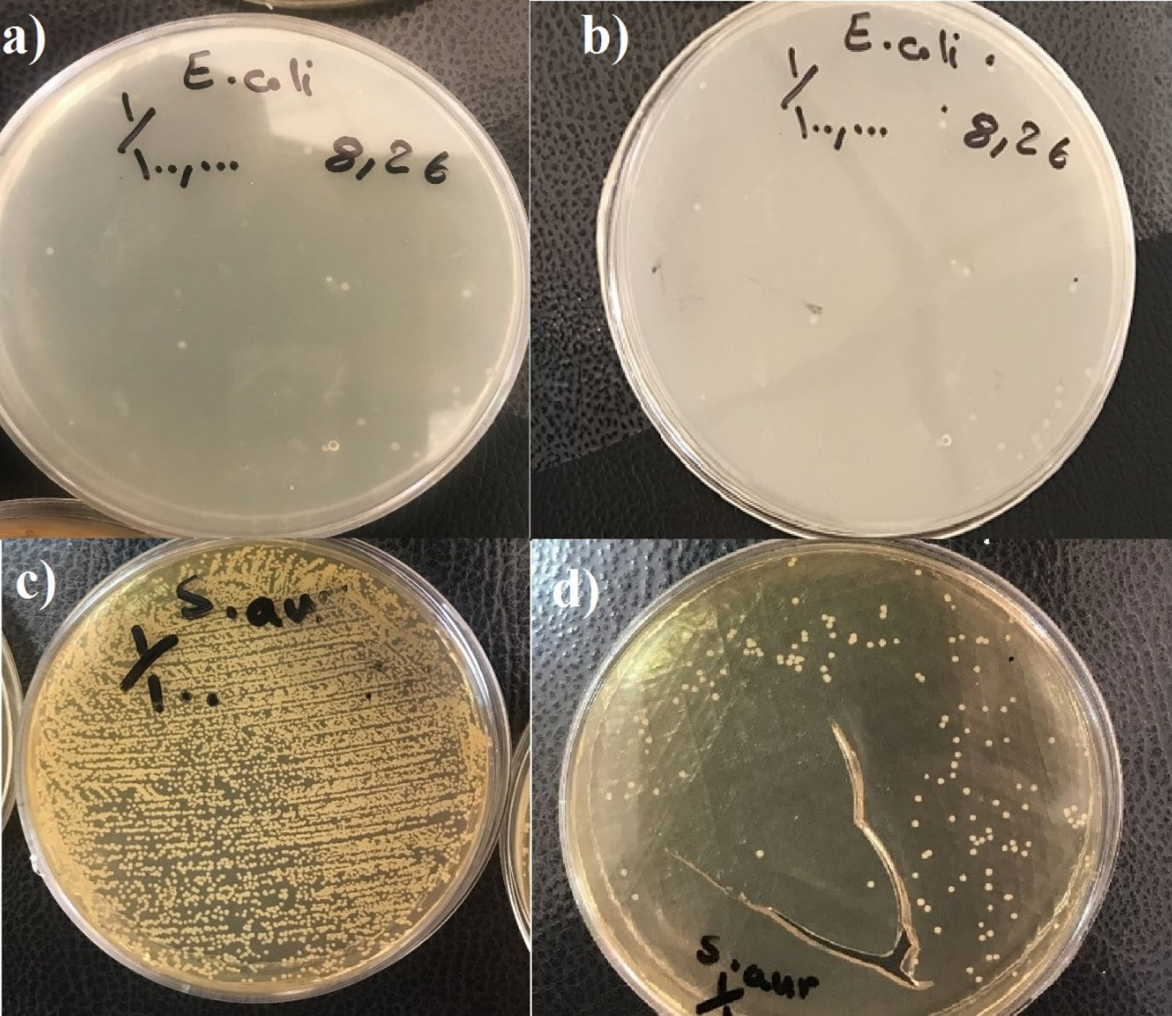
Bacteria growth after exposure to the scaffolds: (**a**) and (**b**) *E. coli*, (**c**) and (**d**) *S. aureus* after treatment with Gel-BG (**a**,**c**) and Gel-BG/sr (**b**,**d**) respectively.

The growth of *E. coli* standard strain ATCC28922 was completely inhibited by each of the bioglass containing scaffolds with no bacterial growth on MH plates. The Gel-BG/Sr partially inhibited the growth of *S. aureus* which resulted in in 1000 colony-forming units (CFU)/mL on MH plates equivalent to 3 log reduction in growth index. However, Gel-BG revealed no antibacterial effect on *S. aureus* as detected by more than 10^6^ (CFU)/mL growth on MH agar (Fig. 6).

## Discussion

Aiming to enhance bone formation with less adverse systemic side effects, Sr has been recently included in many bone substitutes^14^. This ion is a trace element which induces bone development and hinders bone resorption, simultaneously^22–24^. The safety and efficacy of Sr-doped biomaterials for inducing bone formation and remodeling have significantly been reviewed^14^. In our earlier study, we proved that Sr substitution for Ca in Gel/BG scaffolds improves the mechanical, biological, and angiogenic properties of the scaffold^17^. In this experiment, we additionally investigated the osteogenic potential of Gel-BG and Gel-BG/Sr scaffolds in critically sized rabbit calvarial defects. Histological assessments were performed on the harvested scaffolds four, eight-, and 12-weeks’ post-surgery. No chronic inflammation was observed following implantation of the scaffolds, which confirms tissue compatibility of BG containing scaffolds^25,26^. Analysis of decalcified samples with Alizarin red staining presented much more new bone in defects filled with Gel/BG-Sr compared to those contained Gel/BG and the control groups. This indicates that Sr substitution in BG could remarkably increase bone regeneration capacity.

One reason that Sr promotes bone formation and remodeling could be its impact on the expression of genes, including cytokine IL-6. This cytokine, a pro-inflammatory stimulator that recruits osteoclast and induces bone reabsorption, is decreased by Sr effect^27^. Also, Sr induces genes and proteins involved in the bone formation, such as bone morphogenetic proteins and osteocalcin^28^. It has been shown that Sr ions can increase MSCs response as well as to hinder the differentiation of osteoclasts via inhibiting the expression of receptor activator of nuclear factor Kappa-B (RANK) ligand in MSCs^23,29^. Besides, this ion can stimulate osteoprotegerin expression, which in turn stops the RANK and its ligand interaction, inhibiting osteoclast activity^30,31^. It has been demonstrated that the acceleration of osteoprogenitor cells differentiation into osteoblasts could be due to the activation of membrane-bound calcium sensing receptor (CaSR) and the Wnt/β-Catenin signaling pathway^32,33^. Interestingly, Sr has emerged to induce angiogenic factors expression, including vascular endothelial growth factor^30^. Enhanced neovascularization caused by Sr substitution could also provide more nutrients for bone-forming cells in bone defects^19^. Therefore, activation of osteogenesis and angiogenesis could be considered as improving features for Sr containing scaffolds, as has been previously confirmed^19^. Apart from contributing factors mentioned above, surface topography and the synergistic effect of the released bioactive Sr and Si ions from BG is other factors enhancing bone regeneration ability of the Gel/BG-Sr scaffold^19,34^. In a study, it was confirmed that Sr and Si in the structure of BG could synergistically activate the NFATC and Wnt/βCatenin signaling pathways, respectively, which in turn mediate osteogenesis^35^. Zhao et al. assessed the osteogenic capability of Sr-MBG fabricated by three dimensional (3D) printing method in critical-sized defects made in rat calvarial. Sr-MBG scaffolds exhibited superior osteocunductivity and more new vessel formation compared to MBG scaffolds for eight weeks^19^.

The process of bone healing can be delayed due to the bacterial infection, which can subsequently lead to surgical failure by replacement or removal of the implanted biomaterials^36,37^. Therefore, biomaterials with anti-infective properties are required in line with the specific clinical application^38^. Many studies have indicated that BGs, even without ionic additions, have growth-inhibitory influence against several important pathogens^39,40^. Although the exact antibacterial mechanisms for the BGs remain unclear, one possible reason could be the fact that the glass sodium is being released, which is unfavorable for bacteria and increases the pH level. In fact, increased osmotic pressure caused by dissolution of ions, including silicon, calcium, sodium, and phosphate provides an undesirable environment for the bacteria growth. Besides, several activities in the bacterial cell, including glycolysis, trans membrane proton translocation and acid tolerance can be inhibited by dissolution ions such as zinc form BGs depending on the concentration^41,42^. Antibacterial studies of this experiment suggest that strontium substitution could increase the bactericidal effectiveness against *S. aureus* and *E. coli*. The outcome was more pronounced against *S. aureus*. It has been reported that Sr-BGs antibacterial activity could be a result of the higher concentration of Ca, P and Sr ions being released in the simulated body fluid (SBF) solutions and the higher pH values compared to the BG samples^43^. In a study by Liu et al., strontium-substituted BGs significantly inhibited the growth of sub-gingival bacteria depending on the ratio of strontium in the glasses^44^. Interestingly, the authors stated that even the base glass with no Sr displays an apparent antibacterial activity, which may be due to the increased amount of phosphate to 4 mol% compared to the Bioglass 45S5 with 2.5 mol% P_2_O_5_^44^. Significant antimicrobial properties of strontium and silver-containing BG powder has also been formerly confirmed against *S. aureus* and *E. coli* bacteria. Previously, antibacterial test results have shown that the strontium substituted 58S BG could exhibit the antibacterial effect against methicillin-resistant *S. aureus* (MRSA) bacteria which are resistant to methicillin and other associated antibiotics of the penicillin class. Bacterial activities, including growth and reproduction, cell wall synthesis, cell metabolism as well as chromosomal replication can be inhibited by the release of Sr^2+^ ions, as well^43,45–47^.

While Sr doped materials are safe and effective for stimulating bone formation and remodeling, this effect may be more noticeable over time under the concentration applied^14^. Possible factors that could affect the potential evaluation of results for Sr-enriched biomaterials activity are the rate in which Sr is being released, Sr content, experimental animal models, size, location, type of the defects, as well as the applied methods to evaluate the final response^14^. However, despite the good results, more information is required about the safety and effectiveness of local Sr usage^14^. Precautions still need to be considered as the safety of oral Sr ranelate for the cardiovascular system could be a matter of concern^48,49^.

## Conclusion

Bioactivity of biomaterial is an important factor to be considered as it can influence the scaffold effectiveness to induce bone formation18. Inclusion of 15wt% of BG-Sr into a polymer bulk improved its osteogenic ability compared with that containing only BG. Also, Sr incorporation effectively enhanced antibacterial activities against both *S. aureus* and *E. coli*. However, more detailed analysis is still required to elucidate the mechanisms of Sr dopant on improved bone healing for clinical applications.
